# Detecting and Profiling of Milk Thistle Metabolites in Food Supplements: A Safety-Oriented Approach by Advanced Analytics

**DOI:** 10.3390/metabo13030440

**Published:** 2023-03-17

**Authors:** Ancuța Cristina Raclariu-Manolică, Carmen Socaciu

**Affiliations:** 1 Stejarul Research Centre for Biological Sciences, National Institute of Research and Development for Biological Sciences, 610004 Piatra Neamț, Romania; 2 Faculty of Food Science and Technology, University of Agricultural Sciences and Veterinary Medicine Cluj-Napoca, 400372 Cluj-Napoca, Romania; 3 BIODIATECH—Research Center for Applied Biotechnology in Diagnosis and Molecular Therapy, 400478 Cluj-Napoca, Romania

**Keywords:** milk thistle, *Silybum marianum* (L.) Gaertn, silymarin, metabolites, food supplements, quality, safety, advanced analytical approaches

## Abstract

Milk thistle (*Silybum marianum* (L.) Gaertn.) is among the top-selling botanicals used as a supportive treatment for liver diseases. Silymarin, a mixture of unique flavonolignan metabolites, is the main bioactive component of milk thistle. The biological activities of silymarin have been well described in the literature, and its use is considered safe and well-tolerated in appropriate doses. However, commercial preparations do not always contain the recommended concentrations of silymarin, failing to provide the expected therapeutic effect. While the poor quality of raw material may explain the low concentrations of silymarin, its deliberate removal is suspected to be an adulteration. Toxic contaminants and foreign matters were also detected in milk thistle preparations, raising serious health concerns. Standard methods for determination of silymarin components include thin-layer chromatography (TLC), high-performance thin-layer chromatography (HPTLC), and high-performance liquid chromatography (HPLC) with various detectors, but nuclear magnetic resonance (NMR) and ultra-high-performance liquid chromatography (UHPLC) have also been applied. This review surveys the extraction techniques of main milk thistle metabolites and the quality, efficacy, and safety of the derived food supplements. Advanced analytical authentication approaches are discussed with a focus on DNA barcoding and metabarcoding to complement orthogonal chemical characterization and fingerprinting of herbal products.

## 1. Introduction

**Figure 1:**
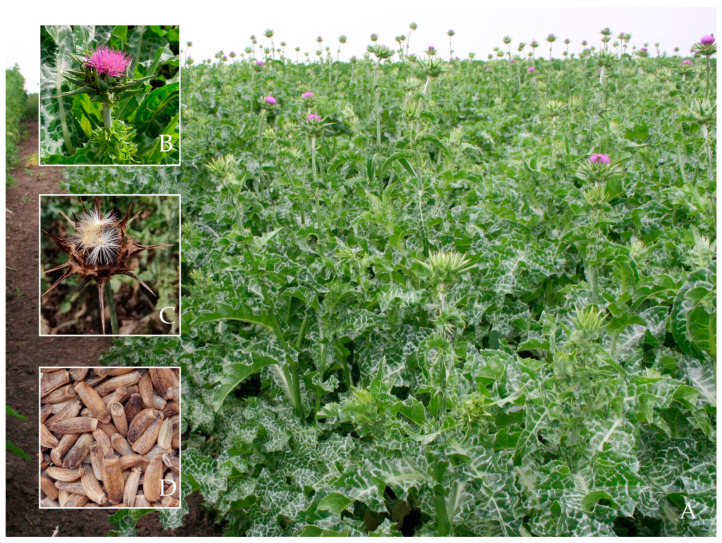
Milk thistle (*Silybum marianum* (L.) Gaertn). (**A**) Cultivated field at the Agricultural Research and Development Station (SCDA) Secuieni, Neamț County, Romania. (**B**) Flower head with spiny bracts and variegated leaf. (**C**) Mature flower (**D**) Fruits (Photos: A.C. Raclariu-Manolică; M. Naie).

*Silybum marianum* (L.) Gaertn. (milk thistle, MT, Figure 1), a member of the *Asteraceae* family, has been used since ancient times as a medicinal plant [1,2,3]. Silymarin, a mixture of unique, antioxidant flavonolignans, is the main bioactive component of MT. Silymarin is indicated to prevent particularly liver and biliary tract disorders and oncological conditions, to support the treatment of hepatitis C, HIV, diabetes, and hypercholesterolemia, as well as to increase lactation [4,5,6,7,8]. MT also has promising potential for other applications ranging from human consumption to industrial exploitation, including the use in dermatological and cosmetic preparations [9,10,11,12]. Although its fruits (achenes), often referred to as seeds (Figure 1D), contain the highest amount of silymarin, other parts of the plant have also been used as traditional remedies [13,14]. Native from Southern Europe, Asia Minor, the Southern Russian Federation, and Northern Africa, MT is spread nowadays in warm and dry regions throughout the world [15], and it is cultivated for the production of silymarin in most areas of Europe, Asia, North and South America, and Southern Australia [10,16,17].

MT-based food supplements are available in a variety of pharmaceutical formulations, including pills, tablets, capsules, or standardized extracts, and can be bought at pharmacies and over largely uncontrolled distribution channels, such as e-commerce platforms [3,18,19]. With attracting claims on their purported health benefits, food supplements are used by consumers with the hope of maintaining their health or supporting specific health conditions. Studies show that up to 43% of patients with liver disease and up to 87% of patients with cancer use hepatoprotective herbal-based food supplements, including MT [20]. The high popularity is spurred, among others, by the consumers’ misconception that botanicals are “natural” and, thus, always “safe” [21]. This assumption and pervasive misleading advertising and poor quality of marketed preparations may lead to serious threats to the consumers’ health and well-being, beyond limitation in reaching the expected beneficial effects.

Inadequate regulatory frameworks and poor quality control strategies are enhancing economically motivated fraud, and a large number of adulterated MT products that include depleted extracts and synthetic colorants were detected in the global market [20,22,23]. MT food supplements were found to have alarming discrepancies between declared and detected chemical content on individual preparations and within batches of the same preparations and manufacturers [19,20,24,25,26]. Most of the investigated preparations contained a significantly lower amount of silymarin than declared on the label of the product, some completely missing silymarin, therefore critically altering the expected therapeutic effects [19,20,24,25,26]. Notwithstanding, MT-derived preparations have been recognized as being safe for human consumption [3,6,27], their widespread contamination with fungi, microbes, and pesticides has been widely reported raising alarming safety concerns for consumer health [20,28]. Some of the studies also reported the presence of foreign matters in the investigated commercial preparations, suspected as being adulterants, but their identity was not determined [19,29,30,31]. Though novel testing and quality monitoring strategies have been recently developed and applied to characterize the composition of MT products and to check for possible adulteration, fraudulent manufacturers and suppliers found ways to deceive such tests [32].

There is a clear need for more robust control of MT food supplements. However, currently, there are some major bottlenecks in ensuring their quality and safety and reducing the potential health risks related to their intake [33,34,35,36,37,38,39]. Since botanical supplements are complex mixtures, using a single comprehensive analytical method for their authentication is challenged by the complexity of these products [40,41,42]. They are, meanwhile, prone to variability under natural conditions that is directly reflected in the batch-to-batch variation of the final composition [43,44]. Moreover, their long and complex supply chains involve ingredients that are often extracted and processed differently, hindering accurate monitoring and quality control processes if only traditional analytical methods are used [44,45,46]. In this context, applying new fit-for-purpose technologies and methodologies will enable a more accurate quality assessment of MT-derived preparations.

While a number of comprehensive reviews discuss the chemical profile, the therapeutical potential of MT, or its potential application in various fields [3,10,47,48], no up-to-date review exists on the MT commercial herbal products authentication. In this respect, our review surveys updated information about the MT constituents and the quality, efficacy, and safety of MT preparations. Furthermore, the review gives an overview of the state of art in MT authentication, underlying the role of innovative analytical approaches to evaluate the overall quality of its commercial preparations.

## 2. Bioactive Metabolites of Milk Thistle

**Figure 2:**
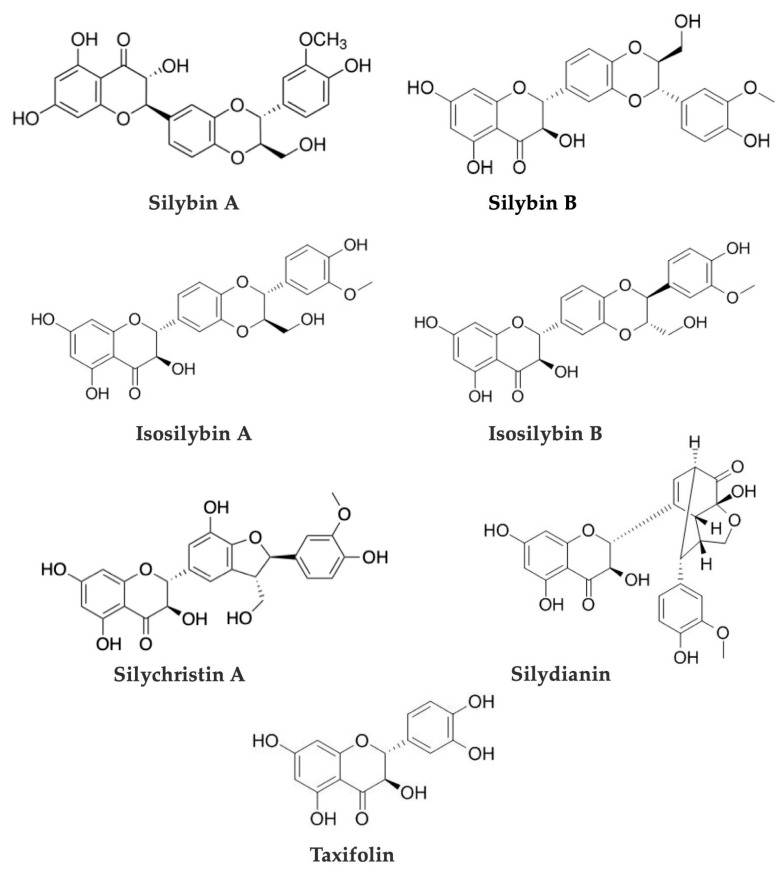
Chemical structures of the main flavonolignans of *Silybum marianum* (L.) Gaertn.

Silymarin is the main bioactive component of MT, a mixture of seven flavonolignan isomers: silybinin (syn. silybin) A and B, isosilybinin (syn. isosilybin) A and B, silychristin A and B, and silydianin (Figure 2) found in leaves, roots, fruits, and mainly in seeds of MT [8,47]. Additionally, the silymarin complex contains as well 30% of a potentially bioactive polyphenolic compound, often referred to as a “polymeric fraction” [49,50]. MT also contains flavonoids (e.g., taxifolin, apigenin, luteolin, naringin, chrysoeriol, eriodyctiol, kaempferol, quercetin, rutin, dihydrokaempferol), fatty acids (linoleic, oleic, palmitic behenic, etc.), sterols (cholesterol, campesterol, stigmasterol, sitosterol), proteins, sugars (arabinose, rhamnose, xylose, glucose) [51]. Silybin is the component with the highest biological activity, representing 50–70% of the silymarin extract, and is the most studied flavonolignan [49,52]. Silybin A, silybin B, and isosilybin A were also detected in cultures of *Aspergillus iizukae*, a fungal endophyte growing on the leaves of MT [53].

The seven major flavonolignans of the silymarin complex and the flavonoid taxifolin are being used as marker compounds for MT authentication and quantitation and tested as recommended by specific monographs, including the European and United States Pharmacopeias, among others [25,47,54,55,56].

Silymarin content in the fruits of MT strongly depends on the MT cultivar, geographical location of cultivation (the physical properties of the soil, weather), and agronomic conditions (time of sowing, fertilizing, irrigation, time of harvest, maturity of seeds) [10,57,58]. Intraspecific variability in the relative silymarin content has been previously reported [10] and may range from 0.62 to 2.25% and exceed 6% in some ecotypes [59]. The effects of cultivated genotypes and environmental conditions on silymarin content in MT plants from different origins (Chilean, German, New Zealand, and Iranian genotypes) have also been reported [60]. In Israel, the polyphenol content was 30% higher in achenes originating from purple inflorescences than those from the white inflorescences [13].

Supplementary Table S1 includes a list of main compound classes and types found in milk thistle seeds and some relevant references. 

Since MT seeds have a high lipid content (20–30%), silymarin extraction in a single step is difficult [47]. Different analytical approaches for optimized extraction of silymarin recommended the use of 20% grounded seeds (as such or defatted) in ethanol 70%, by ultrasonication, or alternative techniques [47]. Wianowska et al. [61] used pressurized liquid extraction to extract silymarin from non-defatted MT fruits and obtained a considerably higher silymarin amount compared with methanol Soxhlet extraction on defatted fruits. Cagdas et al. [62] used ultrasound-assisted extraction of seed silymarin in methanol up to a content ranging from 0.77 to 1.37 g/100 g dried defatted MT. Engelberth et al. [63] separated silymarins by Pressurized Hot Water and fast Centrifugal Partition Chromatography, obtaining extracts with silychristin at 70.2% purity, silydianin at 93.7% purity, and a mixture of silybinin and isosilybinin at 96.1% purity. Bunnell et al. [64] compared various silymarin extraction procedures finding that the ethanol-Soxhlet extraction produced the cleanest High-performance liquid chromatography (HPLC) chromatogram and the highest yield (18.3 mg silymarin/g seed meal), while the Pressurized Hot Water Reactor yielded 5.2 mg silymarin/g seed meal and the Batch Parr reactor 6.2 mg silymarin/g seed. Barretto et al. [65] and Wallace et al., 2003 [66] made the extraction of MT-based nutraceuticals using hot water and organic solvents, respectively. Microwave-assisted extraction has been reported as a simple and efficient method for the extraction of the silymarin [67], and lately, other new methods have also been proposed to increase the extraction yield of the silymarin [68].

The European Pharmacopeia monograph specifies a minimal content of silymarin ranging from 30% to 65% m/M for a dried fruit extract in herbal preparation(s), expressed as silybin, corresponding to the sum of the contents of silycristin and silydianin (20% to 45% of total silymarin), the sum of the content of silybinim A and silybinin B (40% to 65% of total silymarin), and the sum of isosilybinin A and isosilybinin B (10% to 20% of total silymarin) [69]. The United States Pharmacopeia specifies the ripened fruit of MT should contain not less than 2% silymarin, calculated as silybin on the dried matter [70]. The World Health Organization (WHO) specifies that silymarin should be not less than 1.5% in mature fruit, calculated as silybin [15].

## 3. Efficacy and Safety Use of Milk Thistle-Supplements

The silymarin complex is the main responsible for the pharmacological activity of the MT [25,47,54,55,56]. However, individual components contained in silymarin may be selectively responsible for various therapeutical bioactivities [71]. The mechanisms of action of the main bioactive compounds of MT have been discussed by numerous studies, but they are not fully elucidated yet [3,72,73,74]. The effectiveness of MT fruit has been evaluated particularly to test its hepatoprotective role [1,2,75,76,77]. It has been reported as being used for the treatment of drug-induced hepatic cirrhosis and fibrosis and to support the treatment of hepatitis and other chronic inflammatory liver conditions [2,78,79], for stimulation of milk production in lactating mothers [80,81,82], and was investigated for its effects on oncological diseases and metabolic syndrome [2,83,84]. In Europe, intravenous silybinin has been approved as an antidote in patients intoxicated with *Amanita phalloides*, a mushroom that causes fatal poisoning [85].

Generally, the treatment with MT-derived preparations has been recognized as being safe and well-tolerated in recommended doses and specified conditions of use, with a low incidence of adverse effects when compared to placebo [3,6,34,34]. An exceptional case of allergic reactions was reported involving inhalation exposure at high doses of ground milk thistle [86]. Drug interactions are low [48]; only in some circumstances may hepatic metabolism be altered as a result of increased toxicity of certain drugs when co-administrated with silymarin [87]. So far, the paucity of adequate studies does not allow the establishment of a dose-dependency curve for the intake of MT-supplements [6]. Based on the long tradition of use of MT fruit preparations, the Committee on Herbal Medicinal Products (HMPC) of the European Medicines Agency (EMA) released a monograph concluding that they are safe and efficient to be used as medicines with indications for digestive disorders and to support liver function [18].

No report was found, in the European Rapid Alert System for Food and Feed (RASFF) notification system operated by the European Commission (EC), on risks to public health in relation to the consumption of MT supplements [88]. Meanwhile, in VigiAccess, the publicly accessible web-based tool of the World Health Organization (WHO)’s global database VigiBase [89], a number of 1488 reports (on 15.12.2022) were found for the suspected adverse drug reactions of MT as the active ingredient. Most of the reported potential side effects were mild, and they occurred mostly in Asia (69%), followed by Europe (29%). A recent VigiBase study focused on the possible herb-drug adverse reactions when combining chemotherapy with common botanicals, e.g., MT having the highest number of Individual Case Safety Reports [90].

## 4. Commercial Milk Thistle Food Supplements

MT is among the best-selling botanicals in many countries around the world, with a foreseen growing tendency [91,92]. In the global marketplace, MT preparations are available under different regulatory policies, commercial terms, and definitions, including herbal or botanical drugs, traditional or herbal medicines, natural health products, and dietary or food supplements [33,93,94]. In the European Union (EU), MT preparations are regulated and introduced on the market according to their medical or food intended use. The first category (medical use) falls under the EU Directive 2004/24/EC, which establishes a simplified regulatory procedure that allows the registration as a traditional herbal medicinal product (THMP) for the self-medication [95]. The second category, defined as food supplements by the EU Directive 2002/46/EC (European Commission) [96], is regulated by national food laws that are largely not harmonized at the European level [97]. Yet, all food supplements have to comply with the regulatory requirements on their manufacture, hygiene, labeling, nutrition, health claims, composition, testing, storage, and distribution. Food supplements are defined as “foodstuffs, the purpose of which is to supplement the normal diet and which are concentrated sources of nutrients or other substances with a nutritional or physiological effect, alone or in combination, marketed in dose forms namely forms, such as capsules, pastilles, tablets, pills and other similar forms, sachets of powder, ampoules of liquids, drops dispensing bottles and other similar forms of liquids and powders designated to be taken in measures of small unit quantities” [98].

MT food supplements are presented for commercialization under a wide array of formulations as a unique ingredient or in combination with other ingredients in complex matrices [99]. The raw forms of MT are usually commercially available as powdered seeds used for infusions. However, it is important to notice that silymarin has low aqueous solubility, releasing only a small fraction (about 10%) into an aqueous infusion [100,101]. The largest variety of formulations is based on dry extract (standardized) of the MT seeds that are usually processed to extract silymarin [49]. These are largely accessible in the form of capsules, pastilles, tablets, pills, and liquid extracts [102], but also smarter formulations, such as phytosomes and liposomes, developed to increase silymarin bioavailability, which is also found on the market [99,103,104]. Extracts of *Schizandra* berry, artichoke, or dandelion have been reported to be used to enhance the efficacy of MT products [99].

Commercial MT products were reported as being often mislabeled by interchangeable use of the terms flavonoids, silymarin, and silybin [52,105,106]. The incorrectly used nomenclature of the MT-based ingredients raises questions regarding the therapeutical efficacy of the derived preparations [105,106]. Moreover, the information provided on the label regarding the contained ingredients is often heterogeneous and not precise. For instance, some producers claim only the fruit content as ingredients, with no other specification regarding the bioactive compounds, whereas others claim various extracts contents for silymarin, silybin, or flavonoid. A recent report by Pinho et al. [107] evaluated the accuracy of information from the labels of 25 MT-based dietary supplements in Oporto (Portugal) and detected various omissions, errors, and potentially false claims.

## 5. Analytical Approaches Used to Characterize Milk Thistle Formulations

Traditional analytical approaches for the identification and quality control of botanicals and their products, which also applies to MT, have been reviewed before [108,109]. These include a series of procedures based on botanical taxonomy, macroscopic and microscopic examination, chemical analysis performed to ensure the raw plant identity, screening the specific marker compounds (e.g., silymarin), the microbiological purity of the final botanical preparation, as suggested by specific monographs, often by Pharmacopoeias. In addition, for herbal products and preparations, European Medicine Agency (EMA) also specifies the detection of possible toxic constituents, such as heavy metals and toxins, as well as the use of a combination of different chromatographic techniques to detect possible substitutes and adulterants [110].

Phytochemistry-based assays are by far the most commonly used approaches for the identification and quality control of commercial MT preparations. The silymarin flavonolignans, e.g., silybinin (or silybin) A and B, isosilybinin (or isosilybin) A and B, silychristin A and B, and silydianin, together with the flavonoid taxifolin are considered marker compounds used for qualitative and quantitative analyses of MT [25,47,54,55,56]. Some of the methods and recent protocols proposed to check possible contaminants and to evaluate the chemical content of MT products are presented below.

### 5.1. Thin-Layer Chromatography (TLC)

TLC is a cost-effective method for the rapid identification and quality control of both raw plant materials and herbal components in different products [111]. It is recommended for the identification of MT fruits by the European Pharmacopeia [69] and the United States Pharmacopeia [70], both including monographs with specifications and analysis procedures. According to the European Pharmacopeia regarding the identification of MT fruit (*Silybi mariani fructus*), the TLC analysis is applied on methanol extracts (e.g., 1.0 g of powdered drug in 10 mL methanol) in parallel with standards (e.g., 2 mg silybin and 5 mg taxifolin in 10 mL of methanol). The mobile phase is made of anhydrous formic acid: acetone: methylene chloride (volume ratios 8.5:16.5:75). The separated components are visualized with a diphenylboric reagent, under UV light at 365 nm, resulting in a yellowish-green fluorescent band for silybinin and an orange fluorescent band for taxifolin [69].

TLC offers only semi-quantitative data, and it has lower resolution compared with other commonly analytical methods [112] but is useful for a preliminary evaluation of key components from MT.

### 5.2. High-Performance Thin-Layer Chromatography (HPTLC)

HPTLC is the advanced version of classical TLC that allows highly reproducible results and a better separation and detection of the targeted compounds, becoming a valuable tool for the identification of medicinal plants and their derived products [113]. An HPTLC protocol for MT fruits was published by The International association for the advancement of high-performance thin layer chromatography (HPTLC Association) and implemented by the United States Pharmacopoeia Dietary Supplements Compendium [19].

Frommenwiler et al. [19] used HPTLC to investigate 31 MT-based food supplements and traditional herbal medicines. The products were purchased on the internet, in local shops, and in pharmacies, in the United Kingdom and were in various pharmaceutical forms (e.g., tablets, capsules, tinctures, and liquid extracts). Acceptable quality was found in all herbal medicines, while 10 out of 21 food supplements fully complied with the label claims, nine showed the absence of taxifolin position, and two lacked all MT characteristics.

Anthony and Saleh [29] used HPTLC in parallel with high-performance liquid chromatography-mass spectrometry (HPLC-MS) to determine the silymarin composition of 45 products collected from the USA and Egyptian markets. The authors found significant differences in silymarin content (measured vs. declared), including samples having none or very low silymarin levels or even questionable high concentrations of silybin.

### 5.3. High-Performance Liquid Chromatography (HPLC)

HPLC is routinely used to detect and measure the content of targeted bioactive secondary metabolites in herbal products and is recommended as a hyphenated analytical assay for this purpose [114].

The MT monographs for the European Pharmacopeia [58] and the United States Pharmacopeia [59] recommended HPLC as a valuable technique for the identification and quantitation of MT fruits. Wallace et al. [66] developed an HPLC protocol to identify and quantify the concentrations of taxifolin, silychristin, silydianin, silybinin A, and silybinin B of raw MT seed extract and tested this on food supplements purchased from two suppliers in the USA. A high variation of the flavonolignan constituents was found in products vs. seeds, but also between product content and the claimed composition on the label. Kvasnička et al. [115] used HPLC to assess the content and composition of the silymarin components, including silybin, isosilybin, silydianin, and silychristin, in six MT extracts from the European market. The silymarin content ranged between 17.21 to 63.96% and was found to be highly variable among batches made by the same producers.

Ultra-high-performance liquid chromatography (UHPLC) using a core-shell column was used by Fibigr [24] for the separation of taxifolin, silychristin, silydianin, silybin A, silybin B, isosilybin A, isosilybin B from ten commercial samples of herbal teas and food supplements purchased from the Czech Republic. The method showed a high efficacity to separate isomeric compounds and enabled the detection of significant differences in silymarin content (measured vs. declared) in these products.

UHPLC coupled with tandem mass spectrometry (UHPLC-MS/MS) was applied to assess the level of mycotoxin contamination in 32 MT herbal dietary supplements from the Czech Republic, Australia, Costa Rica, Slovakia, France, South Africa, and the USA, including capsules with dried powder/oil-based matrix, seeds, tablets, granules, tea [31]. The authors identified 57 mycotoxins, and the highest mycotoxin level found in MT-food supplements ranged up to 37 mg/kg. Another UHPLC-MS/MS-based protocol was developed by Arroyo-Manzanares et al. [116] to determine mycotoxins in MT, including aflatoxins, fumonisins, trichothecenes, ochratoxin A, citrinin, sterigmatocystin, and zearalenone. The method was validated on seven MT market samples (seeds and extracts), and the presence of 5 mycotoxins, as well as HT-2, T-2, and zearalenone, have been found in some samples. Tournas et al. [117] developed an HPLC method using fluorescence detection (HPLC-FLD) for aflatoxin analysis of 83 MT samples from the USA market. Several aflatoxins were detected in 19% of the samples, with levels ranging from 0.04 to 2.0 µg kg^−1^.

UHPLC coupled with high-resolution mass spectrometry (UHPLC-HRMS) was applied by Fenclova et al. [20] to assess the composition of the main active ingredients and possible contaminants in 26 MT-based food supplements, mainly capsules with dried powder and encapsulated oily paste, purchased from the USA and Czech Republic. The authors found significant differences in silymarin content (measured vs. declared) among individual products but also substantial inter-batch differences in silymarin content and alarming levels of mycotoxins, the presence of various pesticide residues, and microbiological contamination. A total of 37.6 mg/kg of mycotoxin concentration was reported. This is the highest concentration found in MT-based preparations [118], exceeding the concentration of 37 mg/kg reported by Veprikova et al. [28].

Lee et all. [119] developed an HPLC-MS protocol to separate silydianin, silychristin, silybins (silybin A and B), and isosilybins (isosilybin A and B) from commercial MT extracts, finding significant variations of these six constituents.

Simple and fast HPLC protocols with photodiode-array detection (HPLC-DAD) were developed by Petrásková et al. [71] for the rapid determination and quantification of silymarin components and of 2,3-dehydroflavonolignans. The six silymarin preparations showed substantial differences in their composition.

Ultra-high-performance liquid chromatography coupled with quadrupole-time-of-flight mass spectrometry (UHPLC-QTOF-ESI + MS) has been used by Raclariu-Manolică et al. [23] for the authentication of eighteen commercial MT preparations showing significant variations of the detected ingredients.

### 5.4. Direct Spectroscopic Methods

Ultraviolet-visible (UV-Vis) spectroscopy allows a simple and cost-efficient fingerprinting of targeted phytochemicals in plant extracts based on the molecules’ UV and visible light absorption. Near-Infrared and Mid-infrared Spectroscopy is also a fast and noninvasive method often used for quantitative and qualitative analysis of herbal products, particularly in combination with the chemometrics [120]. Ashie et al. [121] developed a rapid quantitative method based on near-infrared spectroscopy (FT-NIR) to determine the active ingredients in MT. Vágnerová et al. [122] also used FT-NIR to determine the content of specific MT components and discriminate among two MT varieties (Silyb and Mirel).

Zavoi et al. [117] used UV-Vis and Infrared (FT-MIR) spectrometry to compare the polyphenolic composition of MT and other medicinal herbs, recommending this approach as being a rapid and reliable tool to investigate the fingerprint and the composition of medicinal plants or to evaluate the quality and authenticity of standardized botanical preparations.

Nuclear magnetic resonance (NMR) spectroscopy is a non-destructive technique used in quality control and research to determine the content and the purity of a sample, as well as its molecular structure [123]. NMR-based untargeted metabolomic was proposed for the discrimination of authentic versus potentially adulterated botanicals [94].

Cheilari et al. [124] compared the performance of the quantitative NMR (qNMR) combined with UHPLC-DAD. The two approaches showed similar performance in quantitating the flavonolignans (silychristin, silydianin, silybin A, silybin B, isosilybin A, and isosilybin B) in the industrial and locally prepared MT extracts.

## 6. DNA-Based Identification and Authentication of Milk Thistle

### DNA Barcoding and Metabarcoding

Twenty years ago, Hebert et al. [125] first introduced the concept of DNA barcoding for species identification using short and standardized regions of the genome, known as “barcodes”. Since then, the concept of standard DNA barcoding has been largely extended and adapted to various needs of the research community, regulatory bodies, and industry regarding the species identification [126]. DNA barcoding revolutionized the identification of the botanical ingredients [127,128], making remarkable contributions to the authentication of herbal products in a quality control and pharmacovigilance context [40,46,129]. British and Chinese national Pharmacopoeias adopted and implemented protocols for using DNA barcoding for species identification, making it applicable as a tool in the industrial quality assurance of herbal products [93,130]. It was used to authenticate commercial herbal products enabling the detection of a large number of adulterated products [131,132]. However, it is important to note that the application of conventional DNA barcoding is limited to the identification of raw material and authentication of single-ingredient herbal preparations before these are undergoing various processing steps that usually lead to the loss, degradation, or mixing of DNA [133].

DNA barcoding combined with high-throughput sequencing (HTS), known as DNA metabarcoding, has the key advantage of enabling multi-taxa identification from complex matrices at any processing or production stage containing DNA from different species [46,134]. Using DNA metabarcoding, large incongruences between the claimed and the detected total species composition were reported by many authors [135,136,137,138]. In a large literature-based survey of data on herbal product authentication, as detected by DNA-based methods, Ichim et al. [132] reported that 27% out of 5957 commercial products sold in 37 countries around the globe were adulterated. Moreover, DNA metabarcoding unlabeled the detection of protected species [135,139] and species with potential concerns for human health (e.g., toxic or allergenic) in a large variety of commercial herbal products [140,141].

The quantification of the relative abundance of a species within the products is beyond the technical capacity of the DNA metabarcoding [131,133]. This narrows its use to the identification of target plant species and confirmation of the presence of possible contaminants and adulterants. To overcome these limitations, DNA metabarcoding has been successfully used together with standardized phytochemical methods to obtain qualitative and quantitative information on the markers of lead chemical compounds. DNA metabarcoding was used together with HPLC-MS to authenticate 16 commercial botanical preparations containing *Veronica officinalis* L. [133], with TLC and HPLC-MS to assess the content of 78 commercial preparations containing *Hypericum* species [137] and with HPTLC to authenticate 53 *Echinacea* herbal preparations sold on the European market [136]. Recently, Raclariu-Manolica et al. [23] used DNA metabarcoding together with Ultra-high-performance liquid chromatography coupled with quadrupole-time-of-flight mass spectrometry (UHPLC-QTOF-ESI + MS) to assess various commercial MT preparations found on the European market.

DNA metabarcoding has the capacity to identify the target species and, at the same time to detect all the other species present within a herbal product that can be possible contaminants or adulterants. This key ability offers DNA metabarcoding a significant potential in the context of post-marketing quality control and pharmacovigilance if used in combination with appropriate analytical chemical methods [133].

## 7. Orthogonal and Complementary Methods for Quality Control

From an analytical perspective, there is still a significant gap regarding the quality control strategies that apply to commercial botanical products, including MT preparations [41,142]. So far, no analytical method used as a stand-alone approach is able to comprehensively address the herbal product authenticity [38,94,109]. Thereby, a combination of complementary and orthogonal analytical techniques is generally recommended to ensure the quality and safety of herbal products [32,46,143,144,145]. Even though definitions for the terms “orthogonal” and “complementary” have been previously proposed in the field of quality control of pharmaceutical and biopharmaceutical products [146], they do not yet have a robust equivalent compatible with the existing analytical methods available for the quality control of herbal products. Following the definition given by Simon Jr. [146], the orthogonal methods are used to measure the same property of a sample in order to reduce the method-specific errors, whereas the complementary methods should be applied to bring more information that reinforces and support a common decision.

Botanical raw materials are complex natural chemical formulations, usually sourced from multiple producers and growers and subject to very different post-harvesting processing, reaching the market as highly processed formulations [44,147]. Moreover, the more complex herbal products contain numerous ingredients hindering the accuracy of traditional analytical methods in identifying the targeted species and, moreover, in detecting the non-targeted species that may occur [44,45].

Apart from the natural and induced complexity of these herbal matrices that are already raising challenges to assess their quality, it has been recently reported that suppliers and manufacturers are aware of the commonly used identification methods and have perfected ways to deceive such tests and to commit various types of fraud [22]. Silymarin is cited as a well-known example of this type of practices [30,32]. In this case, the manufacturers have been shown to make use of spectrophotometric methods for the standardization or obtaining the concentrations declared on the certificates of analysis and labels of the marketed products, even if these methods have been shown to produce a higher value for silymarin (i.e., 30–65% by HPLC-UV vs. 65–80% by spectrophotometric methods) [30,32]. To avoid this heterogeneity of declared information, the method that has been used for the quantification of the targeted compound should always be declared by the manufactures [32]. McCutcheon [30] extracted evidence from the literature showing that 30 to 50% of commercial MT extracts do not comply with their label claims. According to the report, up to 28.6% of MT commercial preparations do not contain silymarin, whereas up to 46% had evidence of adulteration.

The essential role of the new tools in ensuring the safety and quality of botanical products is widely recognized, and the number of studies using it in the context of authentication and quality control of herbal products has lately considerably increased [94]. Simmler et al. [94] and Abraham and Kellogg [147] made comprehensive reviews of integrated analytical approaches applied for botanical product identification and adulteration management discussing that integrated use of targeted and un-targeted analytical tools offers an improved approach to the authentication process of herbal products [94].

The emerging field of plant metabolomics offers new ways to determine the profiles of plant materials which are highly variable under the influence of various factors. Metabolomic approaches have been shown to be accurate, robust, and time-efficient authentication of complex botanical products [147]. Targeted and untargeted metabolomics have been applied to comprehensively investigate chemical variation in various matrices (from plants to food supplements) [148,149,150,151,152].

DNA barcoding and metabarcoding have been applied to assess the total species diversity in processed products, post-marketing control, and pharmacovigilance, and it has been successfully used in several studies related to the quality control of herbal products [132]. Standard DNA barcoding is applicable to detect substitution or adulteration of species only in plant raw material or herbal products containing only a single species and becomes inefficient when the DNA is highly degraded and/or is mixed with the DNA belonging to another species. The great advantage of DNA metabarcoding over traditional DNA barcoding consists in its capacity to simultaneously identify taxa also from multi-ingredient mixtures making this applicable for authentication of complex herbal products containing multiple species [133]. The use of orthogonal chemical characterization and chemical fingerprinting to complement the DNA-based methods has been successfully applied before for the quality control of herbal products [135,136,137,153].

Integrating botanical, macroscopic, sensory, and microscopic-based tests with qualitative or quantitative chemical and spectroscopic techniques assays to determine the identity, purity, and quality of herbal products is considerably increasing the confidence in the results [108]. Moreover, DNA-based methods are particularly important for identifying multiple ingredients in complex mixtures and detecting untargeted species that may be present within the product as possible contaminants or adulterants [133].

## 8. Conclusions and Future Perspective

There is a clear body of evidence that several commercial products claiming to contain MT did not meet their label claims, particularly regarding the bioactive compounds (e.g., silymarin). Various reasons can be behind the low silymarin levels or other ingredients in MT commercial products. While the low quality of raw materials may be one of the causes, the deliberate removal/depletion of the active substances (e.g., silymarin) is suspected to be the main driver [30]. Moreover, the presence of contaminants or suspected adulterants is of alarming concern since the higher incidence of liver injuries was linked to the consumption of botanical preparations contaminated with toxins, e.g., heavy metals, pesticides, or adulterants (other botanicals) or illicit addition of synthetical pharmaceuticals [154,155].

Some studies provided evidence of the presence of undeclared adulterants within commercial MT products, whose identity was not determined [30,31]. The concerns of unreported ingredients used within botanical products may range from simple misleading labeling to potentially adverse drug reactions [156,157,158] or poisoning by contaminants [142]. Thus, these off-label ingredients represent not only commercial fraud but, in certain cases, may generate serious safety concerns [157,159].

The advanced phytochemical methods based on the separation and detection of targeted biomarkers, such as HPLC-MS, NMR, and Infrared Spectroscopy, have a good resolution in quality control and authenticity evaluation of different plants, such as MT and final derived products (herbal medicines or food supplements). However, in some circumstances, such methods are not yet sensitive enough to identify and quantify diverse molecules in complex herbal products and still show a lower resolution in the non-targeted detection of ingredients in mixtures from herbal products [40,133]. Significant progress has been offered in the last years by the metabolomics approach that combines advanced analytics with multivariate statistics [160].

Driven by the increased public demand, the research community, the regulatory authorities, and Pharmacopeias are making high contributions and proposing novel strategies for the identification and authentication of botanicals and their derived preparations in the context of the quality control [38,94,123]. The global regulatory agencies are advocating and supporting the use of orthogonal methods that are fit for purpose and can discriminate closely related species to ensure the quality and safety of herbal medicine and dietary supplements [33].

The integration of DNA barcoding-based and omics-based methods as analytical approaches in authentication and quality control is a promising solution to overcome some limitations of the traditionally used methods for quality control of herbal products. While DNA barcoding and metabarcoding are powerful qualitative methods that focus on ingredient authentication by using standardized fragments of DNA, the metabolomic approach looks at common molecules which can be found either as the key ingredient (to check its presence and quantity in the product) but also as other ingredients (found in plant mixtures of teas, powders of standardized extracts in capsules or tablets, etc.) or other non-declared excipients. Therefore, further work is advisable to validate the use of these new approaches in the quality control context of herbal products, including herbal food supplements containing MT.

## Supplementary Materials

The following supporting information can be downloaded at: https://www.mdpi.com/article/10.3390/metabo13030440/s1, Supplementary Table S1 Different categories and types of compounds found in milk thistle seeds or different herbal formulations and their confirmed activities by different references.
