# Supplementary material for: Detecting and Profiling of Milk Thistle Metabolites in Food Supplements: A Safety-Oriented Approach by Advanced Analytics
Source: Metabolites. 2023 Mar 17;13(3):440. doi: 10.3390/metabo13030440 (PMC10052194; doi:10.3390/metabo13030440)
Supplement: Supplementary file 1 [file metabolites-13-00440-s001.zip › metabolites-2282005-supplementary.pdf]

Supplementary Table S1. Different categories and types of compounds found in milk thistle seeds or different herbal formulations, their confirmed activities by different references.

Category	Compounds	Activity/[references]
Flavonolignans (Silymarin complex)	Silyhermin	Liver protection and regeneration, antioxidant, anti-inflammatory, antitumor/[8,9,16,18, 83, 106, 47]
	Dehydrosilybin	
	Silybin A / B (Silybinin)	
	Silychristin	
	Silydianin	
Phenolic acids	Gallic acid	Antioxidant/[18,23,65]
	Coumaric acid	
	Ferulic acid	
Terpenoids	Coniferyl alcohol	Antibacterial, antifungal, suppression of NO release/[65]
	Carnosic acid	
	Secoisolariciresinol	
Flavonoids	Apigenin	Antioxidant (radical scavenging) /[8 ,9, 18, 23, 65,]
	Naringenin	
	Luteolin	
	Kaempherol	
	Quercetin	
	Taxifolin	
	(Epi)catechin gallate & Epigallocatechin, Epigallocatechin gallate, Catechin 5-glucoside	
	Myricetin	
	Campesterol andbeta-Sitosterol	
	Apigenin 7-glucuronide	
	Luteolin 5-glucoside	
	Rutin	
Fatty acids and lipids	Myristic acid C12:0	Components of cell membranes structure and energetic metabolism in mitochondria/ [47]
	Palmitoleic acid C16:1	
	Palmitic acid C16:0	
	Linolenic acid C18:3	
	Linoleic acid C18:2	
	Oleic acid C18:1	
	Stearic acid C18:0	
	Eicosaenoic acids 20:1, 20:2, C20:3	
	Arachidic acid C20:0	
	Mycolipanolic acid (C24:0)	
	Myristyl linolenate	
	Palmitoylcholines	
	Monoglycerides	
	Sphingosine 1-phosphate	
	Lysophosphatydylchlines 18:2, 20:3	
